# Factors Associated With Newly HIV Infection and Transmitted Drug Resistance Among Men Who Have Sex With Men in Harbin, P.R. China

**DOI:** 10.3389/fpubh.2022.860171

**Published:** 2022-06-02

**Authors:** Shan Hui, Fangfang Chen, Yi Li, Yan Cui, Jinhui Zhang, Ling Zhang, Yisi Yang, Yanlin Liu, Yashuang Zhao, Fan Lv

**Affiliations:** ^1^Department of Epidemiology, College of Public Health, Harbin Medical University, Harbin, China; ^2^Heilongjiang Provincial Center for Disease Control and Prevention, Harbin, China; ^3^Chinese Center for Disease Control and Prevention, Beijing, China; ^4^Jixi Municipal Center for Disease Control and Prevention, Harbin, China; ^5^Harbin Municipal Center for Disease Control and Prevention, Harbin, China

**Keywords:** HIV, MSM, incidence, drug resistance, seroconversion

## Abstract

**Background:**

This study aimed to evaluate HIV incidence, factors associated with HIV incidence and transmitted drug resistance (TDR) among newly infected men who have sex with men (MSM) in Harbin, P.R. China.

**Methods:**

A cohort study was conducted among MSM in Harbin during 2013 and 2018, with a follow-up frequency of every 6 months. Blood samples from MSM were tested for HIV antibodies, RNA was extracted from plasma, and the *pol* gene was sequenced, and genotypic drug-resistance analyses were performed.

**Results:**

From 2013 to 2018, the overall rate of HIV incidence was 3.55/100 PY. Syphilis infection, unprotected sex with men in the past 6 months, and unawareness of HIV/AIDS knowledge were risk factors for HIV seroconversion. The distribution of HIV genotypes was as follows: CRF01_AE, 57.1%; CRF07_BC, 28.5%; CRF55_01B, 2.0%; B, 8.2%. The prevalence of transmitted drug resistance was 4.08%.

**Conclusion:**

HIV incidence in MSM in Harbin is moderately high, and transmitted drug resistance exists in the population.

## Introduction

In 2019, there were 38.0 million people living with HIV and 1.7 million new HIV infections worldwide (1). The proportion of men who have sex with men (MSM) accounted for 12% in 2015 and increased to 23% in 2019 globally (2, 3). In China, the estimated number of people living with HIV was 1.25 million at the end of 2018. The number of MSM accounted for 25.5% of newly identified HIV/AIDS cases in China in 2017 (4). HIV prevalence in MSM increased from 5.73% in 2010 to 6.9% in 2018 in China (3, 5). As a potential bridge population for HIV transmission, HIV epidemic in MSM is of particular concern in China (6, 7).

Longitudinal research is important for tracking the leading edge of the epidemic, monitoring the trend of new infections, and targeting prevention programs (8). However, longitudinal research to estimate HIV incidence, as well as factors associated with HIV infection in MSM in Heilongjiang has not been undertaken. Heilongjiang lies in northeast China. Harbin is the capital city of Heilongjiang province, and the center of MSM gathering. The number of MSM in Harbin accounted for over a half of cumulative identified HIV/AIDS cases across the whole province (9). Most previous studies are cross-sectional studies and focused on the prevalence of HIV in MSM in Heilongjiang province (10). Longitudinal research in MSM is urgently needed to determine the incidence of HIV infection and factors associated with HIV infection.

Antiretroviral therapy (ART) has a prominent effect on reducing mortality in people living with HIV (11). Drug resistance can be transmitted to others, resulting in ART failure (12), which is a challenge for controlling HIV epidemic. TDR is a public health issue as it can affect ART at individual and population level (13). The expansion of ART and pre-exposure prophylaxis should arouse great attention for the issue of TDR. TDR surveillance is of importance to evaluate the spread of HIV drug resistance mutations, and develop antiretroviral therapy strategy (14). Prevalence of TDR in patients of acute HIV infection (AHI) is higher than patients of chronic HIV infections (15). Due to high levels of viremia, the rate of HIV transmission is high during early HIV infection (16). Attention should be paid to the transmission of drug-resistant HIV among MSM because of high-risk behaviors, and high levels of viremia in MSM during the recent infection stage (17). Previous studies have shown that prevalence of HIV TDR in China was low during the last two decades (18–20). Many studies have reported the prevalence of TDR among MSM in China, ranging from 2.4% to 6.3% (21–27). Some articles have reported an increase of TDR among MSM, such as Shanghai and Tianjin (22, 27), some reported a stable TDR, such as Jiangsu and Guangzhou (21, 26). Several studies have been published on the genetic characterization and HIV drug resistance among MSM in Harbin (28–30), although these studies are cross-sectional study. Transmitted drug resistance among newly infected MSM has not been characterized by longitudinal research.

To evaluate the trend of HIV incidence in MSM in Harbin and its risk factors, HIV subtypes, and transmitted drug resistance in newly infected MSM, a prospective survey was conducted in MSM in Harbin, the capital city of Heilongjiang province.

## Materials and Methods

### Study Design and Participant Enrollment

This study was conducted in Harbin between April, 2013 and December, 2018. Men who were 18 years and above, had sex with men in the past 12 months and tested HIV negative at baseline were recruited through snowball sampling at various venues, including the internet, bathing room, sauna, and park, etc.. The participants were followed up every 6 months by trained staff of non-government organizations (NGOs) and collected information on socio-demographic characteristics, history of drug use, sexually transmitted infections (STIs), HIV/AIDS knowledge awareness, and sexual behavior. We defined HIV/AIDS knowledge awareness as correctly responding six or above questions out of eight questions, otherwise defined as unawareness.

### Laboratory Tests

At every follow-up, participants were tested for HIV and syphilis. For HIV testing, we used an enzyme-linked immunosorbent assay (ELISA; Wantai Biotech Inc., China and Lizhu Biotech Inc., Beijing, China) or rapid test (SD; Kyonggi-do Biotech Co. Ltd, Korea and Yingkexinchuang Biotech Inc., Xiamen, China) for screening, and western blotting for confirmation (HIV Blot 2.2; MP Diagnostics, Singapore). For syphilis testing, we used an enzyme-linked immunosorbent assay (ELISA; Yingke-xinchuang Biotech Inc., China) or rapid test (Wantai Biotech Inc., China). Toluidine red unheated serum test (TRUST; Wantai Biotech Inc., China) was used for syphilis confirmation. Positive for both ELISA and TRUST, or positive for both rapid test and TRUST were defined as being infected with syphilis.

RNA was extracted (QIAamp Viral RNA Mini Kit, Germany). Sequences were assembled and edited using Sequencher version 5.0, and aligned using Bioedit, following the manual editing according to HIV-1 reference subtypes downloaded from the Los Alamos HIV Sequence Database. HIV-1 subtypes were classified according to phylogenetic analysis of the *pol* sequence. Phylogenetic analysis was performed using the neighbor-joining method in MEGA version 6.0 for subtyping analysis. Mutations involved with drug resistance were analyzed using the Stanford HIV Drug Resistance Database (hivdb.stanford.edu) (31). Resistance mutations were defined according to the WHO surveillance drug resistance mutation (SDRM) list updated in 2009 (32). Pairwise genetic distances were calculated using the neighbor-joining method based on the Tamura-Nei 93 model, and transmission clusters were defined as pairwise genetic distances of <1.5%.

### Statistical Analysis

The date of HIV incidence was estimated at the midpoint between the date of last HIV negative test and the first HIV positive test. Cox regression model was used to analyze risk factors associated with HIV incidence in univariate and multivariate analyses. Logistic regression model was used to identify risk factors of lost-to-follow-up. Variables with a *p*-value < 0.1 in the univariate analysis were entered into the multivariate models. Cox regression analysis was performed to calculate the hazard ratios (HRs) and 95% confidence intervals (CIs) of interactions between factors on HIV incidence. The effects of combination between factors on HIV incidence were assessed by crossover analysis. All analyses were performed using SPSS 23.0.

## Results

### Baseline Characteristics of the Participants

The median age of the participants was 30 years, 70.6% were single, 22.3% were married or cohabiting with female sex partners, 7.2% were divorced or widowed, 97.5% were of Han ethnicity, 74.5% had received a college education or above, 88.2% had lived in Harbin for more than 2 years, 69.7% found their sex partners through the internet, and 73.3% identified themselves as exclusively homosexual (Table 1).

**Table 1:** Baseline characteristics of the participants.

**Characteristic**		**N**	**(%)**
Age (years)	<20	201	5.7
	20~	1536	43.7
	30~	850	24.2
	40~	925	26.3
Education	Senior high school and below	896	25.5
	College and above	2616	74.5
Marital status	Single	2478	70.6
	Married or cohabiting with female sex Partner	782	22.3
	Divorced or widowed	252	7.2
Place of residence	Local province	3064	87.2
	Other province	448	12.8
Ethnicity	Han	3423	97.5
	Minority	89	2.5
Duration of stay in Harbin	<2 years	416	11.9
	≥2 years	3096	88.2
Main venue for seeking male	Internet	2446	69.7
sexual partners	Public bathroom or sauna	523	14.9
	Park, public toilets or grasslands	338	9.6
	Bar, club, tearoom	205	5.8
Sexual orientation	Homosexual	2574	73.3
	Others	938	26.7

### Incidence of HIV, Factors Associated With HIV Seroconversion

**Figure 1:**
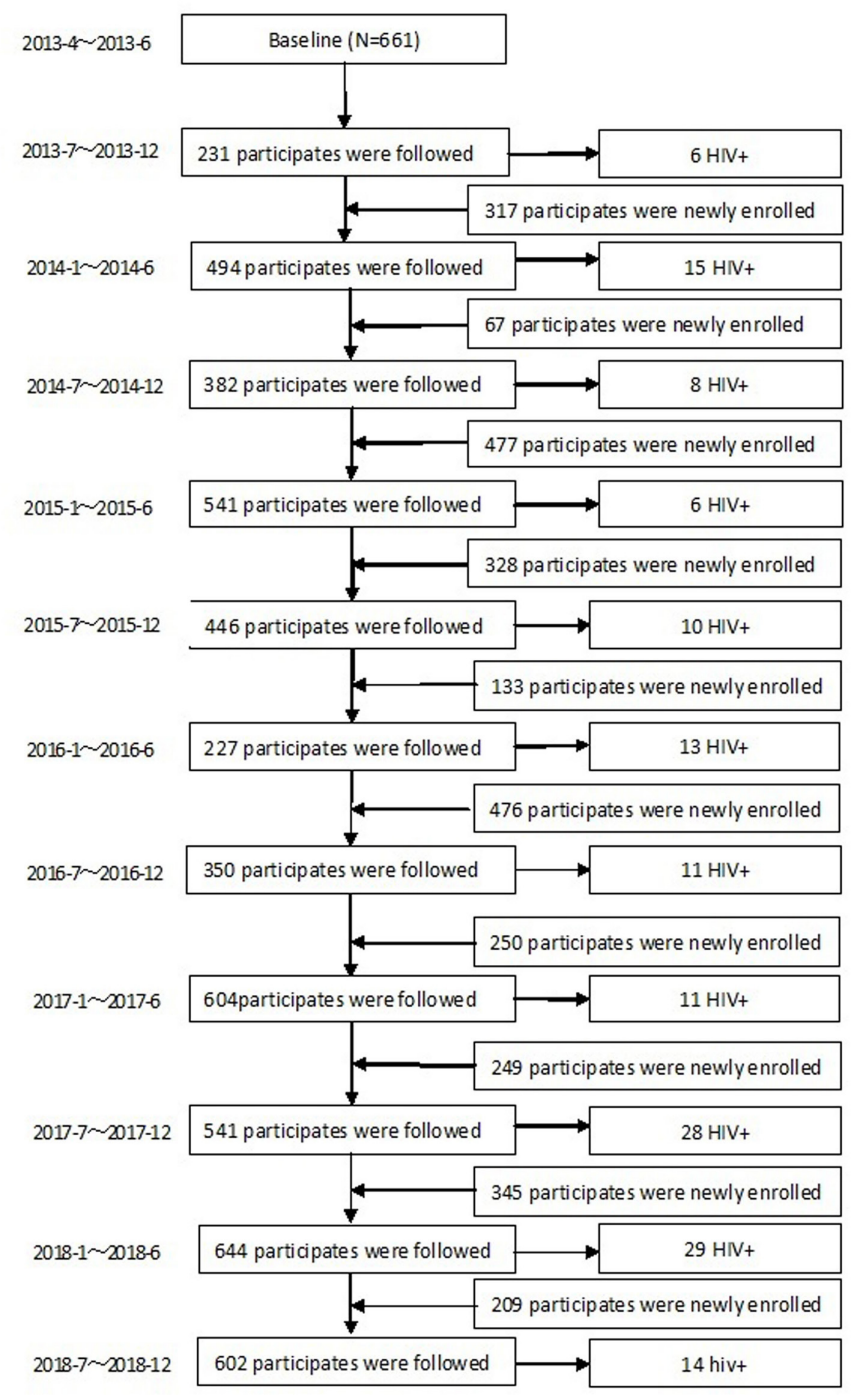
Study flowchart of MSM cohort in Harbin.

During the 68 months of follow-up, 3,512 MSM were enrolled into the dynamic prospective cohort (Figure 1). 80.5% (2,826/3,512) were followed more than once, with a total time of 4,250 person-years (PY). The median follow-up time was 1.31 PY (IQR: 0.63–2.13); 36.9% (1,296/3,512) were followed once, 19.2% (673/3,512) were followed twice, and 24.4% (857/3,512) were followed more than twice. In total, 151 (5.34%) MSM converted to HIV–positive, HIV incidence density was 3.55/100 PY (95% CI: 2.99–4.11/100). HIV incidence in MSM in Harbin decreased from 5.51/100PY (95%CI:3.27–7.75/100PY) in 2013, 3.87/100PY (95%CI:2.57–5.17/100 PY) in 2014, 3.78/100PY (95%CI:2.51–5.05/100PY) in 2015, 2.5/100PY (95%CI: 1.42–3.58/100PY) in 2016, 3.79/100PY (95%CI:2.48–5.1/100PY) in 2017, to 2.35/ 100PY (95%CI:1.04–3.66/100PY) in 2018 (χ^2^ = 5.041, *P*_trend_= 0.025) (**Figure 3**).

Multivariate Cox regression analysis indicated that syphilis infection (HR: 3.90, 95% CI: 2.66–5.72), unprotected sex with males in the past 6 months (HR: 2.71, 95% CI: 1.85–3.98), and unawareness of HIV/AIDS knowledge (HR: 2.57, 95% CI: 1.59–4.13), were independent risk factors for HIV incidence. Having been involved in surveys more than twice was a protective factor (HR: 0.58, 95% CI 0.40–0.84) (Table 2).

**Table 2:** Factors associated with HIV new infection among MSM in Harbin.

**Factors**		**Person years**	**Count**	**Sero-** **conversions**	**HIV incidence**	**Univariate**		**Multivariate**	
						**HR (95% CI)**	***P*** **-value**	**HR (95% CI)**	***P*** **-value**
Main venue for seeking male sexual partners									
	Internet	2486	1937	106	4.26	1.32(0.93–−1.88)	0.116		
	Others	1764	889	45	2.55	1			
Age of sex debut									
	22~	2237	1453	82	3.67	1.11(0.8–−1.53)	0.528		
	<22	2012	1373	69	3.43	1			
Role in sex									
	Bottom or both	2416	1659	100	4.14	1.43(1.02–−2)	0.039	1.41(0.97–−2.06)	0.073
	Top	1834	1167	51	2.78	1		1	
Unprotected sex behavior with male partners in the past6 months									
	Yes	1196	854	82	6.85	2.9(2.01–−4.18)	<0.001	2.71(1.85–−3.98)	<0.001
	No	1963	1233	44	2.24	1		1	
Used additives in the intercourse in the past 6 months									
	Yes	896	626	50	5.58	1.91(1.36–−2.7)	<0.001	1.32(0.90–−1.91)	0.151
	No	3341	2172	95	2.84	1		1	
Times had been followed in surveys									
	>2	3030	1530	71	2.34	0.55(0.4–−0.76)	<0.001	0.58(0.40–−0.84)	0.004
	≤2	1220	1296	80	6.56	1		1	
Syphilis infection									
	Yes	526	317	49	9.31	3.84(2.73–−5.40)	<0.001	3.90(2.66–−5.72)	<0.001
	No	3719	2503	100	2.69	1		1	
Education									
	Senior high school and above	2963	2073	100	3.38	0.78(0.55–−1.09)	0.144		
	Junior high school and below	1287	753	51	3.96	1			
Knowledge of HIV/AIDS									
	No	310	262	28	9.02	2.65(1.76–−3.99)	<0.001	2.57(1.59–−4.13)	<0.001
	Yes	3939	2564	123	3.12	1		1	
The number of sexual partners in the past 6 months									
	<2	2789	1801	105	3.77	0.72(0.45–−1.15)	0.168		
	2~	371	286	21	5.66	1			
Frequency of sexual behavior with homosexual partners in the last week									
	<2	2797	1844	108	3.86	0.78(0.47–−1.28)	0.321		
	2~	363	243	18	4.96	1			
Had sex with commercial sexual partners in the past 6 months									
	Yes	122	86	7	5.74	1.42(0.66–−3.05)	0.366		
	No	3037	2001	119	3.92	1			
Had sex with heterosexual partners in the past 6 months									
	Yes	482	377	22	4.56	1.18(0.75–−1.86)	0.465		
	No	3767	2449	129	3.42	1			

There was a significant combination effect between seeking sex partners through the internet and syphilis infection on the risk of HIV incidence (OR_c_: 6.35, 95% CI: 3.70–10.89). There was a significant combination effect between unprotected sex with male partners in the past 6 months and unawareness of HIV/AIDS knowledge (OR_c_: 5.80, 95% CI: 2.89–11.64). A significant combination effect of unprotected sex with male partners in the past 6 months and syphilis infection on the risk of HIV incidence was observed (OR_c_: 10.89, 95% CI: 6.43–18.45). A significant combination effect between unawareness of HIV/AIDS knowledge and syphilis infection on the risk of HIV seroconversion was observed (OR_c_: 10.10, 95% CI: 4.72–21.58) (Table 3).

**Table 3:** Effects of combination and interaction between factors on the risk of HIV sero-conversion.

**Factors**		**Syphilis infection**				**Unprotected sex behavior with male**		**Having no knowledge of HIV/AIDS**		
						**partners in the past six months**							
		**Yes**	**No**	**Interactions**		**Yes**	**No**	**Interactions**		**Yes**	**No**	**Interactions**	
		${OR}_{c}^{a}$ **(95% CI)**		${OR}_{i}^{a}$ **(95% CI)**	***P*** **-value**		${OR}_{c}^{a}$ **(95% CI)**	${OR}_{i}^{a}$ **(95% CI)**	***P*** **-value**	${OR}_{c}^{a}$ **(95% CI)**		${OR}_{i}^{a}$ **(95% CI)**	***P*** **-value**
Role in sex													
	passive or both	5.67 (3.23–9.94)^*^	1.46 (0.91–2.33)	0.91 (0.41–2.02)	0.821	4.10 (2.17–7.74)^*^	1.60 (0.81–3.16)	0.83 (0.37–1.88)	0.659	3.78 (2.05–6.94)^*^	1.36 (0.9–2.06)	1.26 (0.45–3.54)	0.660
	Initiative	4.25 (2.24–8.09)	1			3.07 (1.57–6.02)	1			2.20 (0.92–5.24)	1		
Used additives in the intercourse in the past6 months													
	Yes	4.91 (2.74–8.8)^*^	1.47 (0.93–2.33)	0.73 (0.33–1.6)	0.429	3.67 (2.11–6.38)^*^	1.78 (0.95–3.35)	0.63 (0.29–1.38)	0.250s	2.11 (0.90–4.90)^*^	1.59 (1.06–2.38)	0.34 (0.12–0.95)	0.039^*^
	No	4.58 (2.74–7.67)	1			3.25 (1.97–5.34)	1			3.92 (2.22–6.95)	1		
Having no knowledge of HIV/AIDS													
	Yes	10.10 (4.72–21.58)^*^	2.63 (1.45–4.79)	0.94 (0.36–2.51)	0.908	5.80 (2.89–11.64)^*^	5.08 (2.46–10.48)	0.35 (0.13–0.91)	0.031^*^				
	No	4.06 (2.60–6.33)	1			3.31 (2.15–5.10)	1						
Main venue for seeking male sexual partners													
	Internet	6.35 (3.70–10.89)^*^	1.34 (0.82–2.19)	1.28 (0.98–1.69)	0.073	3.72 (1.94–7.16)^*^	0.62 (0.37–1.04)	1.17 (0.98–1.39)	0.075				
	Others	2.59 (1.10–6.11)	1			1.27 (0.63–2.55)	1						
Unprotected sex behavior with male partners in the past 6 months													
	Yes	10.89 (6.43–18.45)	2.51 (1.59–3.97)	1.30 (0.56–3.03)	0.547								
	No	3.34 (1.61–6.94)	1										

*${OR}_{c}^{a}$, odds ratio for combining effect of syphilis infection, unprotected sex behavior with male partners in the past 6 months, knowledge of HIV/AIDS and role in sex, used additives in the intercourse in the past 6 months, knowledge of HIV/AIDS, diagnosed as venereal disease in the last year, continuously using condom during sex with male partners in the past 6 months; ${OR}_{i}^{a}$, odds ratio for interaction of syphilis infection, knowledge of HIV/AIDS, unprotected sex behavior with male partners in the past 6 months and role in sex, used additives in the intercourse in the past 6 months, knowledge of HIV/AIDS, diagnosed as venereal disease in the last year, continuously using condom during sex with male partners in the past 6 months. ^a^ adjusted for role in sex, used additives in the intercourse in the past 6 months, knowledge of HIV/AIDS, diagnosed as venereal disease in the last year, unprotected sex behavior with male partners in the past 6 months, syphilis infection, times had been involved in surve;*

* *statistically significant*.

### Factors Associated With Loss to Follow-up

Statistically significant variables in the univariate analysis were entered into a multiple logistic regression model. Having an education of senior high school or above (aOR: 1.32, 95% CI: 1.06–1.64), being married or cohabiting with female sex partners (aOR: 1.66, 95% CI:1.37–2.01), seeking sex partners through the internet (aOR: 1.09, 95% CI: 1.02–1.18), passive role in sex (aOR: 1.59, 95% CI: 1.33–1.91), having not received peer education regarding HIV/AIDS in the past year (aOR: 2.03, 95% CI: 1.64–2.51) were independent risk factors associated with loss to follow-up in the cohort (Table 4).

**Table 4:** Factors associated with loss to follow-up in cohort of MSM in Harbin.

**Factors**		**Follow-up**		**Univariate**		**Multivariate**	
		**Yes (N/%)**	**No(N/%)**	**OR (95% CI)**	***P*** **-value**	**aOR (95% CI)**	***P*** **-value**
Education							
	Senior high school or above	2073(79.24)	543(20.76)	1.38(1.13–1.69)	0.002	1.32(1.06–1.64)	0.015
	Junior middle school	753(84.04)	143(15.96)	1		1	
Ages							
	25~	2028(80.83)	481(19.17)	0.98(0.94–1.03)	0.392		
	<25	798(79.56)	205(20.44)				
Marital status							
	Married or cohabiting with female sex partners	588(75.19)	194(24.81)	1.5(1.24–1.81)	<0.001	1.66(1.37–2.01)	<0.001
	Single, divorced or widowed	2238(81.98)	492(18.02)	1		1	
Main venue for seeking male sexual partners							
	Internet	1937(79.19)	509(20.81)	1.1(1.03–1.17)	0.004	1.09(1.02–1.18)	0.014
	Others	889(83.4)	177(16.6)	1		1	
Role in sex							
	Bottom or both	1659(77.7)	476(22.3)	1.59(1.33–1.91)	<0.001	1.59(1.33–1.91)	<0.001
	Top	1167(84.75)	210(15.25)	1		1	
Having received peer education in the past year							
	No	1139(77.69)	327(22.31)	1.35(1.14–1.6)	<0.001	2.03(1.64–2.51)	<0.001
	Yes	1687(82.45)	359(17.55)	1		1	
Having received HIV test in the past year							
	No	1622(79.16)	427(20.84)	1.22(1.03–1.45)	0.021	1.11(0.93–1.33)	0.235
	Yes	1204(82.3)	259(17.7)	1		1	
Diagnosed as venereal disease in the last year							
	Yes	116(75.82)	37(24.18)	1.33(0.91–1.95)	0.139		
	No	2710(80.68)	649(19.32)				
Have received condom delivery							
	Yes	2026(79.7)	516(20.3)	1.2(0.99–1.45)	0.064	1.64(1.29–2.09)	<0.001
	No	800(82.47)	170(17.53)				
Have used drugs							
	Yes	17(73.91)	6(26.09)	1.46(0.57–3.71)	0.429		
	No	2808(80.5)	680(19.5)				

### HIV Subtype and Transmitted Drug Resistance (TDR)

**Figure 2:**
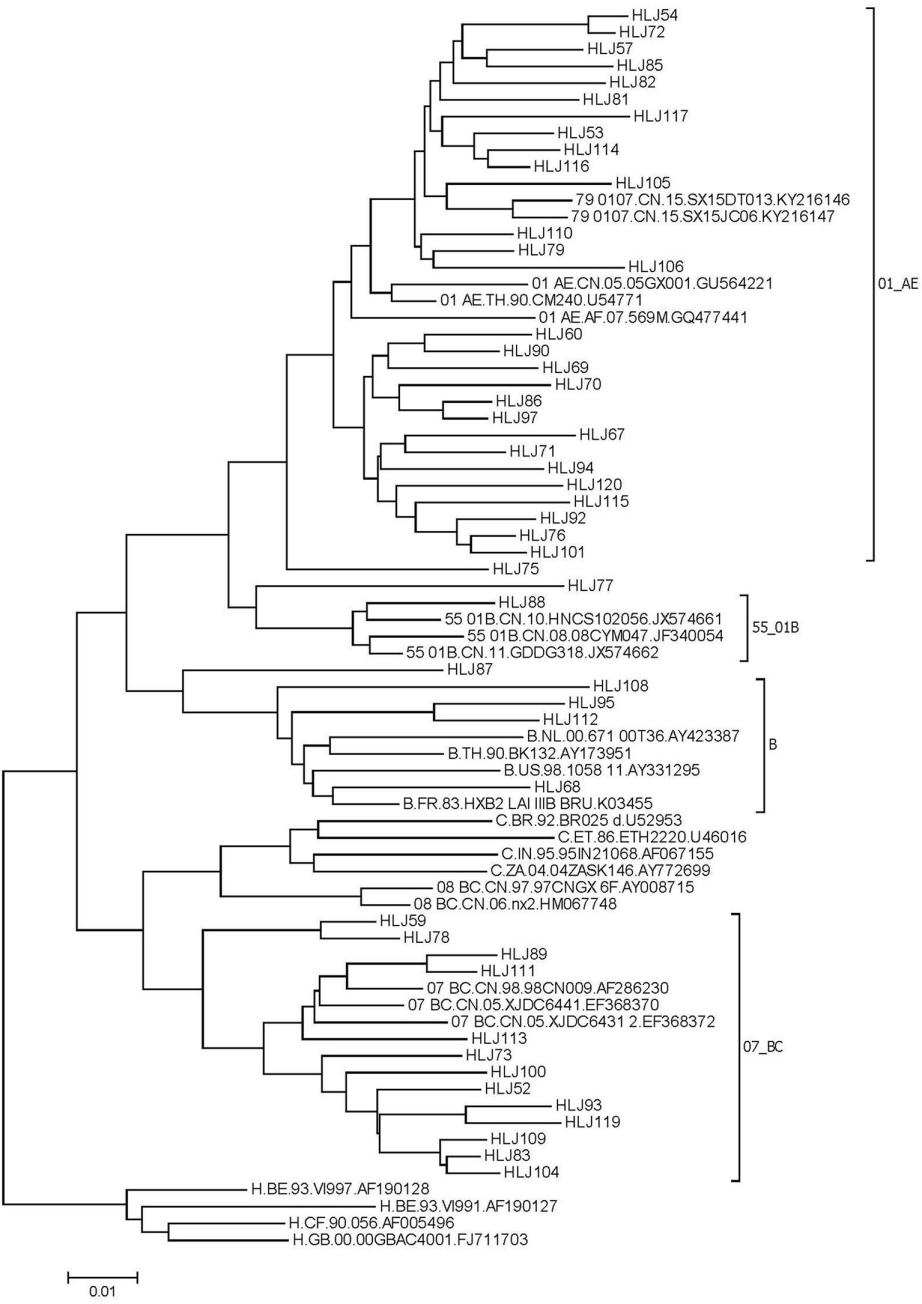
Phylogenetic tree of HIV *pol* gene in MSM in Harbin.

From July, 2016 to June, 2018, 79 new HIV infections were observed, 26 blood samples were not collected, and a total of 53 samples were amplified. Of these, 49 HIV−1 nucleotide sequences were successfully amplified and sequenced. There was no significant difference in age group (χ^2^ = 0^.^561, *P*=0.454), marital status (χ^2^ = 0.434, *P* = 0.51), education (χ^2^ = 0.434, *P* = 0.51), sexual orientation (χ^2^ = 2.736, *P* = 0.098), or cruising venue (χ^2^ = 0.172, *P* = 0.678) between samples successfully amplified and not. The distribution of HIV-1 genotypes was as follows: CRF01_AE, 57.1% (28/49); CRF07_BC, 28.5% (13/49); CRF55_01B, 2.0% (1/49); B, 8.2% (4/49); and URF 6.2% (3/49) (Figure 2).

**Figure 3:**
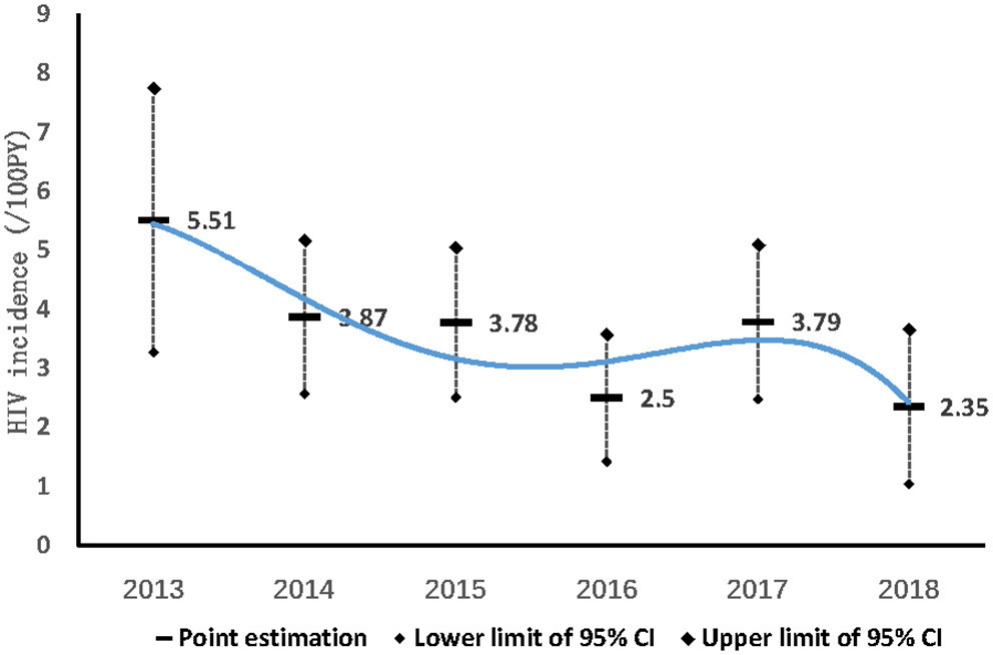
Incidence of MSM in Harbin from 2013 to 2018.

The prevalence of drug resistance was 10.2% (5/49) according to the Stanford HIV Drug Resistance Database, and four mutations in non–nucleoside reverse transcriptase inhibitor (NNRTI) were detected. Two were observed in the subtype CRF01_AE sequence, one was observed in the subtype CRF55_01B sequence, and one was observed in the subtype B sequence. One mutation associated with NNRTI and nucleoside reverse transcriptase inhibitor (NRTI) was observed in the CRF01_AE subtype. Ten mutations, D67G, K70E, Y115F, M184V, V179T, V106M, E138G, K103N, V179E, and V179D, were detected. According to the WHO SDRM list, the prevalence of TDR was 4.08% (2/49). The prevalence of TDR was 2.04% (1/49) for NRTI resistance and 2.04% (1/49) for NNRTI and NRTI resistance.

Nine sequences with genetic distances less than 1.5% were segregated into four clusters. Among them, three clusters were comprised of two individuals, and the other cluster was comprised of three individuals. The clustering rate of CRF07_BC was 23.08%, and that of CRF01_AE was 21.43%. Among the nine individuals, one drug–resistant sample was detected in the molecular clusters. Nine samples in the molecular clusters sought partners through the internet. Four of them had used additive drugs in the past 6 months.

## Discussion

This is the first longitudinal study evaluating HIV incidence and its risk factors, HIV subtype, and TDR in MSM in Heilongjiang province. HIV incidence in MSM in Harbin is 3.55/100 PY, which is consistent with the HIV incidence in MSM in Beijing and Thailand (6, 33), lower than HIV incidence in MSM in Chengdu (7).

In our study, syphilis infection and unprotected sex with men in the past 6 months were independent predictors of HIV incidence, which is similar to factors associated with HIV incidence in Beijing (6). Syphilis facilitates HIV transmission (34), syphilis screening and treatment could be an effective HIV prevention strategy in MSM in Harbin. MSM who were lack of knowledge of HIV/AIDS showed higher HIV incidence, that may because they have less awareness of the dangers of HIV and do not use protection during sexual activities. In addition, there was a significant combination effect between unprotected sex with male partners in the past 6 months and unawareness of HIV/AIDS knowledge (OR_c_: 5.80, 95%CI 2.89–11.64). According to our survey, most MSM sought their partners through the internet. There was a significant combination effect between seeking sex partners through the internet and syphilis infection on the risk of HIV incidence (OR_c_: 6.35, 95% CI (3.70–10.89). In China, along with the continuous development and growing popularity of information technology, an increasing number of MSM seek their partners through the internet. The traditional communication mode of MSM is changing; considering that there are many unknown factors on the internet, behavior intervention strategies in MSM should be adjusted to adapt to this new situation.

MSM who had not received peer education in the past year showed a higher risk of loss to follow–up; to some extent, MSM who have received HIV/AIDS education may be aware of the dangers of HIV and the necessity of regular HIV testing, thus more likely to take HIV testing than those who did not. Participants who were married or cohabiting with female sex partners showed a higher risk of loss to follow–up, which may be a result of the fact that MSM receive more pressure from their family or wife, are more secretive, and have fewer chances for HIV testing.

Genotypic analysis found that 57.1% of samples were CRF01_AE and 28.5% were CRF07_BC among newly HIV–infected MSM in Harbin, which is consistent with the genotypic analysis in Kunming MSM in China and the distribution of HIV genotypes in a cross–sectional study of HIV subtypes in Heilongjiang province (29, 30).

The TDR of HIV in MSM in Harbin was 4.08%, consistent with the studies among MSM in China (3.1%), Kunming (5%), Guangxi (4.8%), Jiangsu (4.0%), Guangdong (3.32%) (18, 21, 24–26, 35), lower than the TDR among MSM in Tianjin (6.3%) (27), but higher than the TDR among MSM in Xi'an (2.4%) (23). The prevalence of HIV−1 TDR was relatively low among MSM in Heilongjiang province, which suggests that current strategies of treatment are effective in MSM in Heilongjiang province. Prevalence of mutations related to NNRTI resistance were higher in the study, which might be attributed to lower genetic barriers to NNRTI resistance, or the use of first–line regimen (18, 36). In our survey, the major HIV genotype in MSM in Harbin was CRF01_AE, accounting for half of the samples. It has been reported that CRF01_AE infection is associated with rapid CD4+ T–cell decline and more likely progression to AIDS than infections of other types (37, 38). TDR in CRF01_AE infections should be considered. In our study, the TDR rate of HIV new infections in the MSM cohort from Harbin was low, although samples with drug–resistant mutations were present in the molecular clusters with a high risk of transmission, which suggests that TDR surveillance in MSM is necessary.

This study had some limitations. First, the retention rate of our survey was relatively low, and the incidence of HIV may be biased because it is not clear whether the HIV incidence of the participants retained in the survey were the same as those lost to follow–up, the representativeness of MSM may be limited. Second, 79 MSM seroconverted to HIV positive during the follow–up period from 2016 to 2018, while only 49 HIV−1 nucleotide sequences were successfully amplified. Although there was no significant difference in demographic characteristics between samples successfully amplified and not, the results may be biased in evaluating the distribution of HIV−1 genotypes among newly HIV–infected MSM in Harbin. The third limitation was the use of Sanger sequencing for genotypic drug resistance genes, which is unable to detect variants at levels below approximately 20% (39, 40). Next–generation sequencing for the detection of transmitted drug resistance mutations should be performed in future research. The fourth limitation was that pooled nucleic acid amplification testing (NAAT) was not used for the screening of HIV–negative participants and samples in the HIV acute infection stage could not be detected (41). If acutely infected participants who cannot be detected by ELISA are lost to follow–up, HIV incidence may be underestimated. The fourth limitation was, we could not analyze the relationship between socioeconomic characteristics such as occupation and income and HIV incidence for lack of information which need to be further explored.

## Conclusion

Above all, HIV incidence of MSM in Harbin is moderately high. Syphilis infection, unprotected sex with males in the past 6 months, and lack of knowledge of HIV/AIDS were independent predictors of HIV incidence. The transmitted drug resistance rate of HIV new infections in the MSM cohort in Harbin was low, although samples with drug-resistant mutations were present in the molecular clusters with a high risk of transmission. Surveillance of TDR should be conducted. In our survey, most MSM sought their partners through the Internet. Considering that there are many unknown factors on the internet, behavioral intervention strategies in MSM should be adjusted to adapt to this new situation, and early identification and effective interventions are urgently needed to break the transmission of HIV in MSM in Harbin.

## Data Availability Statement

The datasets used and/or analyzed during the current study are available from the corresponding author on reasonable request.

## Ethics Statement

The studies involving human participants were reviewed and approved by this study was approved by Institutional Review Board of National Center for AIDS/STD Control and Prevention, Chinese Center for Disease Control and Prevention (Project NO: X130419285). The patients/participants provided their written informed consent to participate in this study.

## Author Contributions

YZ, FL, SH, and FC conceived and designed. SH, and JZ performed the experiments. SH, FC, and YL analyzed the data. YZ, FL, SH, and FC wrote the paper. All authors contributed to the article and approved the submitted version.

## Funding

Project of research on new HIV infection in MSM, National Center for AIDS/STD Control and Prevention, Chinese Center for Disease Control and Prevention.

## Conflict of Interest

The authors declare that the research was conducted in the absence of any commercial or financial relationships that could be construed as a potential conflict of interest.

## Publisher's Note

All claims expressed in this article are solely those of the authors and do not necessarily represent those of their affiliated organizations, or those of the publisher, the editors and the reviewers. Any product that may be evaluated in this article, or claim that may be made by its manufacturer, is not guaranteed or endorsed by the publisher.
